# NRF2 signalling in environmental metal and metalloid neurotoxicity: distinguishing stress responses from functional neuroprotection

**DOI:** 10.3389/ftox.2026.1988769

**Published:** 2026-10-26

**Authors:** Bolaji Samuel Mesole, Uthman Ademola Yusuf, Alfred Omachonu Okpanachi, Rademene Sunday Oria, Flavien Nsoni Bumbangi

**Affiliations:** 1 Human Anatomy Unit, Department of Medicine and Clinical Sciences, School of Medicine, Eden University, Lusaka, Zambia; 2 Department of Human Anatomy, School of Medicine and Health Science, Mulungushi University, Livingstone, Zambia; 3 Department of Medicine and Clinical Sciences, School of Medicine, Eden University, Lusaka, Zambia; 4 Department of Human Anatomy, University of Cross River State, Calabar, Nigeria

**Keywords:** environmental metals, mitochondrial dysfunction, neuroinflammation, neurotoxicity, Nrf2, oxidative stress

## Abstract

Exposure to toxic metals and metalloids is an important public health concern because they can damage the nervous system and impair cognitive and motor function. Oxidative stress, disturbed glutathione balance, mitochondrial dysfunction and neuroinflammation are common features of this damage. Nuclear factor erythroid 2-related factor 2 (NRF2) regulates proteins that help cells maintain redox balance and withstand toxic stress. Increased NRF2 expression after metal exposure is therefore often interpreted as evidence of protection. However, expression alone does not show that the pathway is working effectively. Functional activation requires NRF2 nuclear translocation, binding to antioxidant-response elements, induction of target genes and improvement in redox or mitochondrial function. This review critically examines human, animal and cellular evidence concerning NRF2 responses to cadmium, lead, mercury, arsenic and manganese. It considers how dose, duration, brain region and cell type shape these responses and evaluates compounds intended to modify NRF2 signalling. It assesses whether NRF2 dependence was demonstrated using genetic deletion, knockdown, pharmacological inhibition or rescue rather than inferred from expression changes. Current evidence supports NRF2 as an important but context-dependent defence mechanism. However, causal evidence remains uneven, human data are limited and many proposed activators have effects beyond NRF2. Future studies should use environmentally relevant exposures and directly measure pathway activity, mitochondrial function and neurological outcomes. This is necessary to determine whether NRF2 modulation can provide safe and meaningful protection against environmental neurotoxicity.

## 1 Introduction

Exposure of humans to hazardous metals and metalloids remains a major public health issue. Lead exposure occurs in battery manufacture and recycling and through lead-containing paint, contaminated dust and drinking water (ATSDR, 2020). Fish and other seafood are major dietary sources of methylmercury (World Health Organization, 2024), whereas arsenic exposure often occurs through contaminated groundwater and food (World Health Organization, 2022). Cadmium exposure occurs through food, tobacco smoke and industrial emissions (ATSDR, 2012a); manganese exposure occurs through food, water and occupational inhalation (ATSDR, 2012b). Repeated exposure and slow elimination can prolong internal metal burden (Tchounwou et al., 2012). Human co-exposure to manganese and lead has been associated with altered neurodevelopment (Claus Henn et al., 2012). Age and nutritional status can also influence internal exposure: iron deficiency increases cadmium absorption (Flanagan et al., 1978), and brain-barrier transporter expression changes during development (Verscheijden et al., 2020).

The brain is particularly vulnerable to oxidative injury because of its high energetic demand, dependence on mitochondrial oxidative phosphorylation and abundance of oxidisable lipids (Halliwell, 2006; Cobley et al., 2018).

Environmental metals can intensify this vulnerability through several interacting mechanisms. Depending on their chemical properties, they may bind to cellular thiols, displace essential metal ions, weaken glutathione-dependent defences, disturb intracellular calcium homeostasis and impair the mitochondrial electron transport chain (Cheng et al., 2021). Mitochondrial dysfunction is of particular importance since it can establish a self-sustaining cycle of declining adenosine triphosphate production, increased reactive oxygen species generation and defective mitochondrial quality control (Cheng et al., 2021; Pickles et al., 2018). As this cycle progresses, neurons become less able to maintain synaptic activity and cellular homeostasis. The resulting metabolic stress may also activate microglia and other inflammatory pathways, ultimately promoting synaptic dysfunction, neuronal injury and cell death (Sarkar et al., 2018; Li et al., 2025).

NRF2 is a major regulator of cellular responses to oxidative and electrophilic stress. Its stabilisation and nuclear translocation activate antioxidant-response-element-regulated genes involved in glutathione metabolism, thioredoxin defence, detoxification and cellular repair (Baird and Dinkova-Kostova, 2011; Yamamoto et al., 2018).

NRF2 has effects beyond the induction of antioxidant enzymes. NRF2 can affect mitochondrial substrate availability, membrane potential, respiration and adenosine triphosphate production (Holmström et al., 2013). Loss of NRF2 impairs several of these processes, whereas genetic or pharmacological activation can improve mitochondrial bioenergetics under appropriate conditions (Holmström et al., 2013; Dinkova-Kostova and Abramov, 2015). NRF2 can also restrain inflammatory signalling in microglia and other immune cells, thereby linking redox regulation with the control of neuroinflammation (Innamorato et al., 2008). These actions make NRF2 an attractive mechanistic and therapeutic target in metal-induced neurotoxicity.

Nevertheless, an increase in NRF2 expression after metal exposure does not, by itself, demonstrate effective protection. It may reflect a successful adaptive response, an inadequate attempt to restore cellular homeostasis or an accumulation of NRF2 protein that does not result in productive transcription (Yamamoto et al., 2018; Dodson et al., 2019). Evidence of nuclear translocation, binding to antioxidant response elements, induction of canonical target genes and measurable restoration of redox or mitochondrial function provides a stronger basis for concluding that the pathway has been functionally activated. Even these observations establish association rather than causation. Evidence for NRF2 dependence is more convincing when a protective effect is weakened or abolished by genetic deletion, gene silencing or selective pharmacological inhibition (Ni et al., 2010; Ye et al., 2016; Li et al., 2025). Confidence is strengthened further when genetic and pharmacological approaches produce convergent findings. This distinction is important because oxidative, mitochondrial and neurological injury may continue despite an apparent increase in NRF2 abundance (Xiong et al., 2021).

Previous reviews have examined NRF2 biology in the central nervous system (Heurtaux et al., 2022) and NRF2 responses to toxic metals across different tissues and organs (Buha et al., 2021; Tripathi et al., 2024). However, the literature on metal-induced neurotoxicity has not been consistently evaluated using a framework that distinguishes NRF2 abundance from functional pathway activation and causal NRF2 dependence. This review therefore integrates evidence from human studies, animal models and cellular experiments involving cadmium, lead, mercury, arsenic and manganese. It examines how dose, exposure duration, brain region and cell type shape NRF2 responses and evaluates whether reported pharmacological protection is genuinely dependent on NRF2. The central aim is to determine when NRF2 provides meaningful neuroprotection, when its increased abundance is primarily a marker of cellular stress, and what evidence is required before NRF2 modulation can be considered a credible therapeutic strategy.

## 2 NRF2 signalling in the nervous system

Under basal conditions, NRF2 is maintained at low levels through KEAP1–CUL3-mediated ubiquitination and proteasomal degradation (Yamamoto et al., 2018). Oxidative or electrophilic stress modifies KEAP1, allowing NRF2 to accumulate, translocate to the nucleus and associate with small MAF proteins, where it binds antioxidant-response elements and induces cytoprotective genes (Itoh et al., 1997; Yamamoto et al., 2018). Key NRF2-regulated genes include NQO1, GCLC, GCLM, HMOX1 and TXNRD1, which collectively support glutathione metabolism, thioredoxin defence, detoxification and cellular redox homeostasis (Hayes and Dinkova-Kostova, 2014). However, these genes are not induced uniformly across cell types or exposure conditions; therefore, increased NRF2 abundance or expression of a single target such as HMOX1 should not be regarded as sufficient evidence of functional pathway activation.

NRF2 also contributes to mitochondrial and metabolic homeostasis. In addition to supporting glutathione, thioredoxin and NADPH-dependent defence, NRF2 influences mitochondrial substrate availability, membrane potential, respiration and ATP production (Holmström et al., 2013; Dinkova-Kostova and Abramov, 2015). More recent experimental evidence further indicates that NRF2 regulates glucose uptake and partitioning between energy production and antioxidant defence in neurons and astrocytes, with cell-type-specific metabolic effects (Esteras et al., 2023). These observations reinforce the importance of including mitochondrial and metabolic outcomes when determining whether NRF2 activation is biologically effective.

NRF2 activity differs substantially among nervous-system cell types. Mature neurons generally exhibit substantially lower NFE2L2 expression and chromatin accessibility than non-neuronal support cells, a pattern recently confirmed across multiple mouse and human central-nervous-system datasets (Bell et al., 2015; Levings et al., 2023). Astrocytes exhibit comparatively strong NRF2-dependent antioxidant and metabolic capacity and can support neighbouring neurons through glutathione-related and metabolic mechanisms (Shih et al., 2003; Sun et al., 2005; Esteras et al., 2023). Microglia also use NRF2 to constrain oxidative and inflammatory responses, linking redox regulation with neuroimmune control (Innamorato et al., 2008). Thus, changes in NRF2 measured in whole brain or mixed-cell preparations cannot necessarily be attributed to neurons.

NRF2 can directly restrict transcription of selected pro-inflammatory cytokine genes, as demonstrated in macrophages (Kobayashi et al., 2016). Its reciprocal interaction with NF-κB links antioxidant and inflammatory signalling (Wardyn et al., 2015). Interactions with PGC-1α connect NRF2 to mitochondrial biogenesis (Gureev et al., 2019), while kinase signalling and autophagy provide additional routes of pathway regulation (Yamamoto et al., 2018). These cell-specific and pathway relationships are summarised in Figure 1.

**FIGURE 1:**
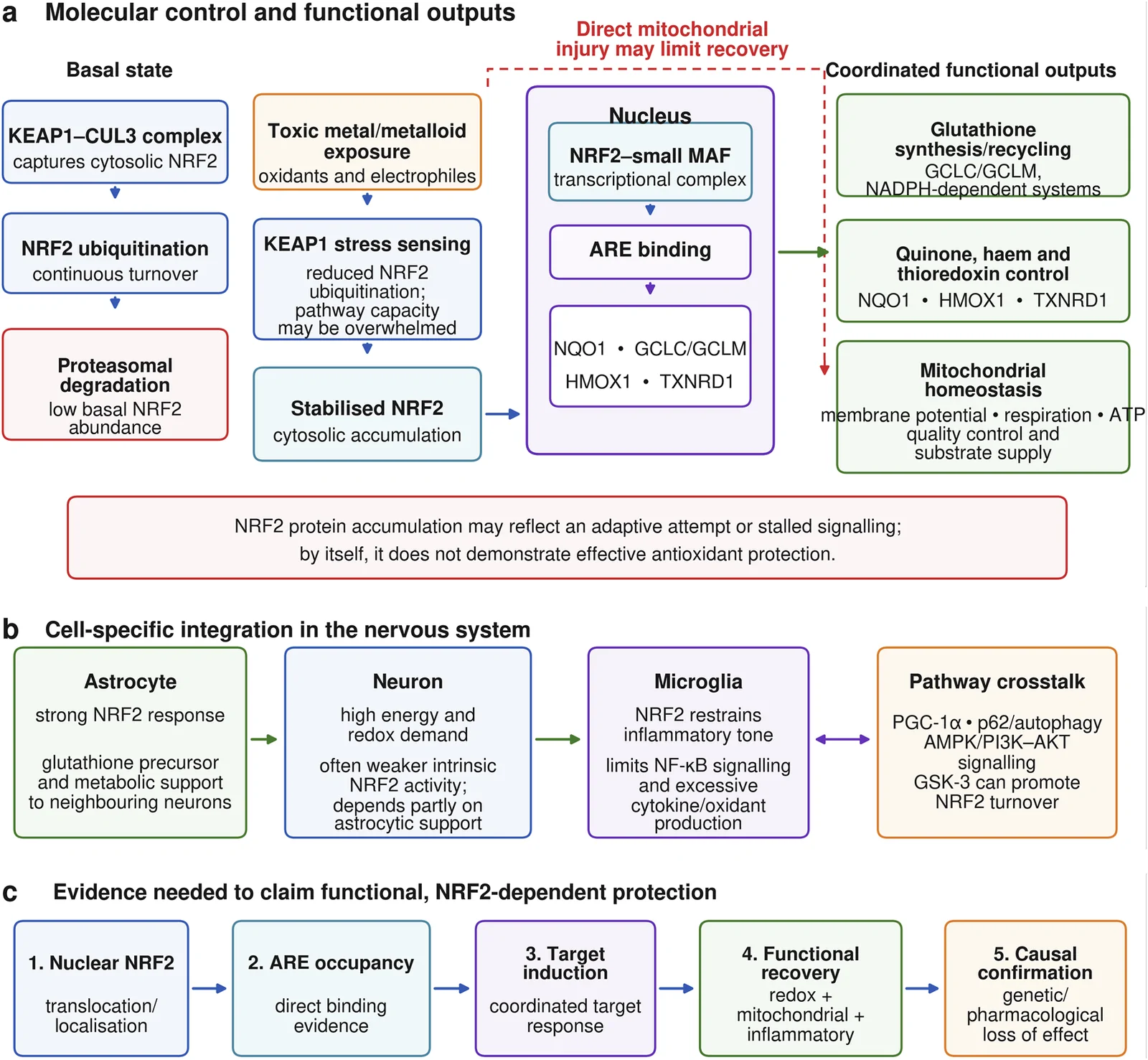
Functional NRF2 signalling in the nervous system during toxic-metal exposure. **(a)** Molecular control and functional outputs. Under basal conditions, the KEAP1–CUL3 complex promotes NRF2 ubiquitination and proteasomal degradation, maintaining low cytosolic NRF2 abundance. Toxic-metal- or metalloid-associated oxidative and electrophilic stress can impair KEAP1-dependent NRF2 turnover, allowing NRF2 stabilisation, nuclear translocation, heterodimerisation with small MAF proteins and binding to antioxidant-response elements (AREs). Subsequent transcriptional induction of NQO1, GCLC/GCLM, HMOX1 and TXNRD1 contributes to glutathione synthesis and recycling, quinone detoxification, thioredoxin-dependent redox regulation and mitochondrial homeostasis. However, direct mitochondrial injury may limit functional recovery despite NRF2 activation, and increased NRF2 abundance alone does not establish effective antioxidant protection. **(b)** Cell-specific integration in the nervous system. Astrocytes exhibit substantial NRF2-responsive antioxidant capacity and provide metabolic and glutathione-related support to neighbouring neurons, which have high energetic demands and comparatively limited intrinsic NRF2 responsiveness. In microglia, NRF2 contributes to the regulation of inflammatory signalling, including NF-κB-associated responses and excessive oxidant production. Pathway interactions involving PGC-1α, p62/autophagy, AMPK/PI3K–AKT and GSK-3 further influence NRF2 signalling and turnover. **(c)** Evidence required to establish functional NRF2-dependent protection. Mechanistic evaluation should integrate nuclear NRF2 localisation, direct evidence of ARE occupancy, coordinated induction of downstream targets and demonstrable recovery of redox, mitochondrial and inflammatory functions. Genetic or pharmacological loss-of-function approaches provide stronger causal confirmation that the observed protection depends on NRF2 signalling.

The principal NRF2-regulated proteins, their nervous-system functions and the evidence required to demonstrate functional pathway activation are presented in Supplementary Table S1.

## 3 Exposure, toxicokinetics and entry into the nervous system

Human exposure to neurotoxic metals and metalloids occurs in occupational and environmental settings. Mining, smelting, battery manufacture and recycling can release lead and cadmium (ATSDR, 2012a; 2020), while welding is an important source of inhaled manganese (ATSDR, 2012b). Workers may inhale metal-containing dusts, fumes or vapours (Tchounwou et al., 2012). General-population sources include lead-contaminated paint, household dust and drinking water (ATSDR, 2020); cadmium in food and tobacco smoke (ATSDR, 2012a); methylmercury in fish and seafood (World Health Organization, 2024); arsenic in groundwater and food (ATSDR, 2007); and manganese in food, drinking water and contaminated air (ATSDR, 2012b).

Ingestion and inhalation are the principal routes of exposure, whereas dermal absorption is generally limited for most inorganic metal compounds (Tchounwou et al., 2012). Route nevertheless depends on chemical form and exposure circumstances. Young children are particularly susceptible to lead exposure because frequent hand-to-mouth and object-to-mouth behaviour increases their ingestion of contaminated paint, soil and household dust (ATSDR, 2020). Children also absorb a greater proportion of an ingested lead dose than adults (World Health Organization, 2026).

Absorption, distribution and persistence differ substantially among metals and their chemical species. Methylmercury is efficiently absorbed from the gastrointestinal tract (ATSDR, 2024). Gastrointestinal absorption of cadmium is generally lower but increases with iron deficiency (Flanagan et al., 1978). Cadmium accumulates mainly in the liver and kidneys; metallothionein binding and slow excretion contribute to biological half-lives measured in decades (ATSDR, 2012a). Lead is initially distributed in blood and soft tissues before substantial deposition in mineralised tissues (ATSDR, 2020). Bone becomes a long-term reservoir from which lead can be remobilised during pregnancy, lactation, ageing and other conditions involving increased bone turnover (ATSDR, 2020).

Inorganic arsenic is metabolised through reduction and methylation reactions and is eliminated predominantly in urine (ATSDR, 2007). It is generally cleared more rapidly than cadmium or skeletal lead, although continuous exposure can maintain a persistent internal burden (ATSDR, 2007). Manganese differs from the other metals because it is an essential nutrient. Under physiological conditions, its absorption and tissue distribution are regulated, and excess manganese is eliminated mainly through the hepatobiliary system (ATSDR, 2012b). These controls may be overwhelmed during sustained environmental or occupational exposure. Inhalation is particularly important because it partly avoids the regulatory processes that limit gastrointestinal manganese absorption (ATSDR, 2012b).

Metals do not enter the brain through a single common mechanism. The methylmercury–cysteine complex resembles methionine and crosses the blood-brain barrier through the L-type large neutral amino-acid transport system (Kerper et al., 1992; Azevedo et al., 2023). Manganese can enter through several pathways whose relative contributions depend on oxidation state, concentration and binding to plasma proteins. Experimental evidence supports both saturable and non-saturable components of manganese uptake across brain barriers, involving free manganese ions as well as protein-bound species (Murphy et al., 1991; Rabin et al., 1993).

The olfactory system may provide an additional route following inhalation. In rats, intranasally administered manganese initially accumulated in the olfactory bulb and subsequently progressed through secondary and tertiary olfactory pathways into other parts of the brain and spinal cord (Tjälve et al., 1996). Cadmium also entered the anterior olfactory bulb after intranasal administration but did not progress extensively beyond the primary olfactory pathway (Tjälve et al., 1996). These results support the biological feasibility of nose-to-brain transport, particularly for manganese. However, the quantitative importance of this route in environmentally or occupationally exposed humans remains uncertain and should not be inferred directly from experiments involving intranasal instillation in rodents.

Metal entry and injury at brain barriers should be distinguished. Lead transport across cerebral endothelium has been demonstrated in rat perfusion experiments (Deane and Bradbury, 1990). Cadmium can also impair blood-brain barrier integrity: rat studies identified altered permeability and reduced microvascular antioxidant capacity (Shukla et al., 1996), while cellular experiments demonstrated disruption of the tight-junction protein ZO-1 (Branca et al., 2019). Barrier disruption can therefore facilitate further exposure, but it should not be treated as the sole explanation for metal accumulation in the brain.

Distribution within the nervous system is also uneven. Manganese characteristically accumulates in basal ganglia structures, particularly the globus pallidus, although deposition can extend into the striatum, white matter and cortical regions during sustained exposure (ATSDR, 2012b; Criswell et al., 2012). Metals entering through the olfactory system may initially be concentrated in the olfactory epithelium and bulb (Tjälve et al., 1996). Methylmercury is distributed more widely throughout the brain (ATSDR, 2024). During prolonged exposure, some methylmercury is demethylated to inorganic mercury, which can be retained in brain tissue considerably longer than the parent compound, as demonstrated in non-human primates (Vahter et al., 1994).

Age, nutritional status and concurrent exposures further modify metal toxicokinetics. Iron deficiency can increase gastrointestinal cadmium absorption (Flanagan et al., 1978), whereas lower iron and calcium status has been associated with greater lead burden in children (Wang et al., 2012). Age-related differences in blood–brain and blood-cerebrospinal-fluid barrier transporters may also influence the distribution of metals within the developing nervous system (Verscheijden et al., 2020; Wu et al., 2023). Co-exposures to metals can have additive, antagonistic or synergistic effects on uptake and toxicity rather than predictably additive effects (Rai et al., 2010; Claus Henn et al., 2012).

Human biomonitoring should be interpreted in relation to the metal, chemical species and sampling matrix. Blood lead reflects circulating exposure and lead released from tissue stores (ATSDR, 2020), whereas urinary cadmium can reflect accumulated body burden, subject to the influence of renal injury (ATSDR, 2012a). Consequently, a peripheral exposure measurement should not be treated as a direct measure of metal concentration in the brain.

These toxicokinetic considerations are relevant for the interpretation of experimental studies. Manganese transport across brain barriers involves regulated and concentration-dependent pathways, with foundational experimental studies demonstrating saturable components of uptake (Murphy et al., 1991; McCabe and Zhao, 2021), and cadmium can disrupt endothelial tight-junction integrity (Branca et al., 2019). High, short-term exposures may therefore engage processes that do not reproduce chronic environmental exposure. Findings should therefore be evaluated in relation to exposure route, chemical species, dose, duration and measured metal burden in the relevant brain region. Exposure intensity and duration can also shape the NRF2 response, helping to determine whether increased NRF2 reflects an effective adaptation, an insufficient response to continuing injury or merely an indicator of metal-induced cellular stress (Pi et al., 2003; Janasik et al., 2018; Xiong et al., 2021).

## 4 Evidence for NRF2 disruption by selected metals

### 4.1 Cadmium

Cadmium illustrates why NRF2 abundance should not be treated as a simple marker of antioxidant protection. The intact blood-brain barrier limits cadmium entry (ATSDR, 2012a). Experimental cadmium exposure can nevertheless compromise microvascular antioxidant defences and increase barrier permeability (Shukla et al., 1996).

Cadmium does not generate reactive oxygen species through classical Fenton chemistry. Its oxidative effects arise mainly from binding to protein and low-molecular-weight thiols, depleting glutathione, displacing essential metal ions and weakening antioxidant enzymes (ATSDR, 2012a). Cadmium also stimulates NADPH oxidase, disturbs calcium regulation and promotes mitochondrial oxidant production (El-Kott et al., 2020; Xu et al., 2016). In rat hippocampus, cadmium reduced glutathione and manganese superoxide dismutase while increasing lipid peroxidation, NADPH oxidase activity, tumour necrosis factor-α, interleukin-6 and NF-κB activation (El-Kott et al., 2020). Glutathione depletion is particularly important because it removes a major thiol buffer while limiting the substrate available for glutathione peroxidase-dependent peroxide removal.

Reported NRF2 responses are not consistent across models. Some studies describe reduced NRF2 or nuclear NRF2, increased KEAP1 and lower HO-1 expression in the hippocampus following repeated cadmium exposure (Srivastava et al., 2023). By contrast, another rat study observed persistent NRF2 nuclear translocation despite continuing oxidative injury, impaired autophagy and apoptosis; protection by trehalose was accompanied by reduced, rather than increased, nuclear NRF2 (Tang et al., 2019). These apparently opposing findings probably reflect differences in dose, duration, brain region, cell type and sampling time. More importantly, increased nuclear NRF2 during unresolved injury may represent an unsuccessful compensatory response. Most cadmium studies measure total or nuclear NRF2 and HO-1, whereas direct antioxidant-response-element occupancy and coordinated induction of NQO1, GCLC, GCLM and TXNRD1 are rarely examined.

Mitochondrial injury provides a functional test of whether this response is adequate. In hippocampal HT-22 cells, cadmium inhibited oxidative phosphorylation and glycolysis, increased mitochondrial superoxide and reduced ATP generation (Poteet et al., 2014). Cadmium also caused mitochondrial fragmentation, membrane-potential loss and ATP depletion in cortical neurons (Xu et al., 2016). These changes are consistent with the memory, learning and olfactory deficits reported after environmentally relevant exposure in mice (Wang et al., 2018).

Quercetin treatment has been associated with improved hippocampal NRF2-related signalling and cognitive performance following cadmium exposure (Srivastava et al., 2023). However, such compounds act through several pathways, so these associations do not establish NRF2 dependence. Stronger evidence comes from NRF2-knockout mice and NRF2-knockdown microglia, in which cadmium caused greater microglial activation, synaptic engulfment and learning-memory impairment (Li et al., 2025). Nevertheless, cadmium neurotoxicity studies combining a defined NRF2 activator with selective pharmacological inhibition, ARE binding, target-gene induction and mitochondrial functional measurements remain limited.

Human evidence provides an epidemiological context for these experimental findings. In US adults aged 60 years or older, higher blood cadmium was associated with poorer cognitive performance; the cross-sectional design could not establish causality or determine whether NRF2 dysfunction mediated the association (Li et al., 2018).

### 4.2 Lead

Lead neurotoxicity is particularly important during development because lead crosses the placenta, accumulates in bone and can be remobilised long after external exposure has declined (ATSDR, 2020).

Circulating lead can enter the central nervous system across brain barrier interfaces. Rat perfusion studies demonstrated lead transport across the cerebral endothelium of the blood-brain barrier (Deane and Bradbury, 1990). In contrast, *in-vitro* choroid-plexus studies showed that early lead exposure reduced the integrity of the blood-cerebrospinal-fluid barrier (Shi and Zheng, 2007). Although both interfaces may influence lead distribution within the brain, they are anatomically and functionally distinct.

Lead is not strongly redox-active under physiological conditions, but it promotes oxidative injury indirectly. It binds cellular thiols, disrupts calcium and zinc homeostasis, depletes glutathione and impairs the activities of superoxide dismutase, catalase and glutathione peroxidase (ATSDR, 2020). Its influence on NRF2 is context dependent. Depending on the dose, duration and experimental model, increases in NRF2-related proteins may coexist with persistent oxidative injury, whereas prolonged exposure may impair p62/KEAP1/NRF2 signalling and weaken downstream antioxidant defence (Peng et al., 2025). Therefore, increased total NRF2 or a single target protein should not be interpreted as evidence of effective antioxidant protection. Lead also increases mitochondrial oxidant production, impairs respiratory chain activity, decreases mitochondrial membrane potential and ATP generation and promotes permeability transition and mitochondria-mediated apoptosis (Qu et al., 2020). These effects provide a mechanistic basis for disturbances in learning, attention and memory. In children, blood lead has been associated with reduced intellectual performance, including at low exposure levels (Lanphear et al., 2005). In adults, past occupational lead exposure has been associated with subsequent cognitive decline (Schwartz et al., 2000).

Stronger evidence of NRF2 involvement comes from experimental treatment with tert-butylhydroquinone, which increased NRF2 nuclear localisation, antioxidant-response-element binding and the expression of NQO1, GCLC and HMOX1 (Ye et al., 2016). Knockdown of NRF2 or HMOX1 attenuated these protective effects, supporting NRF2 dependence within the experimental models studied (Ye et al., 2016). Nevertheless, these findings remain preclinical and should not be interpreted as evidence that tert-butylhydroquinone is an established treatment for human lead poisoning.

Longitudinal human evidence also indicates that childhood lead exposure may have lasting consequences: higher blood lead at 11 years of age was associated with lower IQ at age 38 years in a New Zealand cohort (Reuben et al., 2017). However, among adults newly employed at lead-recycling plants, changes in blood lead over 2 years were not significantly associated with changes in the cognitive tests assessed after full covariate adjustment (Yu et al., 2021). These studies address different exposure windows and populations, and neither establishes a role for NRF2 in the observed neurological outcomes.

### 4.3 Mercury

Mercury cannot be considered a single toxicant because its toxicokinetics and neurotoxicity depend strongly on its chemical form. Elemental mercury is encountered mainly as vapour and, following inhalation, is readily absorbed through the lungs and crosses the blood-brain barrier and placenta (ATSDR, 2024).

Within tissues, elemental mercury is oxidised to divalent inorganic mercury, which crosses membranes less readily and can be retained in the brain (ATSDR, 2024). Inorganic mercury salts are poorly absorbed from the gastrointestinal tract and primarily affect the kidneys (ATSDR, 2024). Experimental barrier models show lower transfer of inorganic than organic mercury species (Lohren et al., 2016). Methylmercury is the organic form of greatest dietary concern because it bioaccumulates and biomagnifies in fish and seafood (ATSDR, 2024).

Methylmercury is efficiently absorbed from the intestine and readily reaches the developing and adult brain (ATSDR, 2024). After binding to L-cysteine, it forms a complex resembling methionine that is transported by the L-type large neutral amino-acid transporter LAT1, facilitating cellular uptake and passage across biological barriers (Yin et al., 2008). Methylmercury readily reaches the developing brain and can disrupt neurodevelopment (ATSDR, 2024).

Methylmercury binds avidly to thiol-containing molecules, including glutathione, and to selenol groups in antioxidant selenoproteins (ATSDR, 2024). These interactions reduce glutathione availability, disturb the GSH/GSSG balance and weaken antioxidant defence (Ni et al., 2010). In primary microglia, acute exposure rapidly increased oxidant production, reduced the GSH/GSSG ratio and promoted NRF2 nuclear translocation, followed by increased expression of Hmox1, Nqo1 and Slc7a11, which encodes xCT (Ni et al., 2010). NRF2 knockdown reduced target-gene induction and increased cell death, demonstrating NRF2-dependent adaptive cytoprotection in this acute cellular model, although not complete or sustained protection (Ni et al., 2010).

Mitochondria are also important targets. In rat striatal synaptosomes, methylmercury-induced mitochondrial depolarisation was associated with disrupted calcium regulation (Dreiem and Seegal, 2007). In human embryonic neural progenitor cells, nanomolar methylmercury exposure reduced mitochondrial membrane potential and ATP content and promoted apoptosis (Wang et al., 2016). Thus, NRF2 activation may represent an adaptive response without complete functional protection. Direct evidence of NRF2 dependence in neural injury caused by elemental or inorganic mercury remains limited.

In young adults, higher hair mercury was weakly associated with poorer performance on tasks requiring rapid processing and with differences in hippocampal, thalamic and white-matter structure (Takeuchi et al., 2022). These observational findings support the relevance of mercury exposure to human neurological outcomes but do not establish NRF2-mediated injury or protection.

### 4.4 Arsenic

Human exposure of greatest toxicological concern involves inorganic arsenic, principally arsenite and arsenate; common organic arsenic compounds in seafood are generally less toxic and more rapidly eliminated (Naujokas et al., 2013). Accordingly, urinary arsenic measurements require chemical speciation to distinguish inorganic arsenic and its metabolites from seafood-derived organic arsenic, which can elevate total urinary arsenic without indicating an equivalent exposure to inorganic arsenic (ATSDR, 2007).

Arsenate [As(V)] is reduced to arsenite [As(III)], which undergoes AS3MT-catalysed methylation (Thomas et al., 2007). Glutathione influences these methylation reactions (Ding et al., 2012). Although methylation facilitates elimination, trivalent methylated intermediates can be highly cytotoxic (Stýblo et al., 2000); methylated trivalent arsenicals also induce oxidative DNA damage in cultured cells (Nesnow et al., 2002).

The NRF2 response to arsenic varies with dose and exposure duration. During relatively low or short-term exposure, arsenite can modify redox-sensitive cysteine residues in KEAP1 and increase hydrogen peroxide signalling. This allows NRF2 to accumulate in the nucleus and stimulate antioxidant response element-regulated genes (Pi et al., 2003). Such activation may transiently enhance glutathione synthesis and cellular detoxification. However, activation of NRF2 *per se* does not mean protection of the brain. For example, exposure to sodium arsenite activated hippocampal NRF2 pathway in mice, but still associated with oxidative injury and impaired spatial memory (Xiong et al., 2021). With more intense or prolonged exposure, glutathione may become depleted, antioxidant enzymes may be inhibited, and NRF2-mediated protection may become insufficient. Evidence from occupationally exposed populations also suggests that chronic arsenic exposure may impair the NRF2-KEAP1 pathway (Janasik et al., 2018).

Mitochondrial dysfunction appears to be an important contributor to this progressive loss of protection. Chronic arsenite exposure reduces the activity of respiratory complexes I, II and IV, decreases mitochondrial superoxide dismutase activity and increases mitochondrial protein and lipid oxidation (Prakash et al., 2015). The associated loss of membrane potential and ATP production can impair synaptic function and promote neuronal apoptosis. Early development is particularly vulnerable because arsenic can cross the placenta (ATSDR, 2007). In a cohort of 1,006 mother-child pairs, prenatal inorganic arsenic exposure, especially during early pregnancy, was associated with poorer mental and psychomotor development at 2 years of age, even in a relatively low-exposure population (Chen et al., 2023). In a cross-sectional study of rural-dwelling adults, higher groundwater arsenic exposure estimated using a geographic information system was associated with poorer language and executive functioning, with domain-specific associations varying across AS3MT genotypes (Edwards et al. 2014). These findings provide epidemiological context for arsenic-associated cognitive impairment but do not establish causality or demonstrate NRF2 involvement.

### 4.5 Manganese and other relevant metals

Manganese is an essential nutrient, but excessive exposure can damage the nervous system, particularly when inhalation bypasses gastrointestinal regulation or hepatobiliary elimination is impaired (ATSDR, 2012b).

Manganese accumulates preferentially within the basal ganglia, especially the globus pallidus, putamen and caudate nucleus. Increased signal intensity in these regions has been demonstrated by T1-weighted magnetic resonance imaging in exposed welders (Criswell et al., 2012). Sustained accumulation can produce manganism, characterised by slowed movement, rigidity, dystonia, postural instability and changes in mood or behaviour (ATSDR, 2012b).

Manganese can disturb mitochondrial calcium regulation, respiration and ATP production (ATSDR, 2012b). In astrocytes, manganese-induced disruption of mitochondrial dynamics has been linked to inflammatory responses (Sarkar et al., 2018). These findings place astrocytic mitochondrial injury among the processes that may contribute to manganese neurotoxicity.

NRF2 responses differ between cell types. Astrocytes generally maintain stronger basal NRF2 activity and glutathione production than neurons (Shih et al., 2003; Bell et al., 2015). Manganese can increase astrocytic NRF2 expression, but it may simultaneously reduce DJ-1, a protein that helps stabilise NRF2, making the overall response difficult to interpret (Lee et al., 2012). In dopaminergic-like PC12 cells, manganese increased NRF2 nuclear accumulation, antioxidant-response-element binding and HMOX1 expression (Li et al., 2011). Pharmacological NRF2 activation reduced apoptosis, although NRF2 dependence was not established by selective inhibition or gene deletion (Li et al., 2011). Other metals also interact with redox and NRF2 pathways, although the strength of evidence varies by metal and experimental system (Buha et al., 2021).

Although the magnitude and direction of NRF2 responses differ according to metal, exposure duration, experimental model and brain region, the available evidence demonstrates convergence on glutathione disruption, mitochondrial dysfunction and neuroinflammation. The principal findings are summarised in Table 1.

**TABLE 1:** Evidence linking environmental metal exposure to altered NRF2 signalling and neurotoxicity.

Metal	Study/model	Exposure	Brain region or cell type	NRF2-related outcome	Redox or mitochondrial outcome	Neurological outcome	Major limitation
Cadmium	Montes et al. (2015); developing rats	Acute CdCl_2_ exposure	Cortex, striatum, hippocampus, cerebellum and glial cells	Increased NRF2 immunoreactivity; involvement inferred (I)	Regional cadmium accumulation; mitochondrial function not measured	Minimal acute histological changes	No nuclear translocation, ARE binding, target-gene induction or NRF2 loss-of-function assessment
Lead	Ye et al. (2016); developmental rats and SH-SY5Y cells	Developmental lead exposure with tBHQ intervention	Hippocampus, frontal cortex and neuronal cells	Nuclear NRF2, ARE binding and HMOX1, NQO1 and GCLC induction; dependence demonstrated in cells (D)	Increased GSH and SOD; decreased lipid peroxidation and apoptosis	Reduced lead-induced neural injury	NRF2 dependence was established in cultured cells but not genetically in the intact animal; tBHQ has additional actions
Mercury	Ni et al. (2010); primary microglia	Acute methylmercury, 1–5 µM	Primary microglial cells	NRF2 nuclear translocation and induction of *Hmox1*, *Nqo1* and *Slc7a11*; NRF2 knockdown increased cell death (D)	Increased oxidant production and decreased GSH/GSSG ratio	Microglial injury and death	Short, micromolar exposure; no neuronal, behavioural or intact-animal outcome
Arsenic	Xiong et al. (2021); mice	Sodium arsenite, 50 mg/kg twice weekly for 7 weeks	Hippocampus	Increased NRF2/PPARγ expression and antioxidant proteins; activation supported but dependence not established (S)	Increased ROS despite induction of SOD, HMOX1 and GPX3	Impaired spatial learning and memory	High parenteral dose; no ARE-binding or NRF2 loss-of-function experiment
Manganese	Li et al. (2011); PC12 cells	MnCl_2_ for 24 h, with or without tBHQ	Dopaminergic-like PC12 cells	Nuclear NRF2 accumulation, ARE binding and HMOX1 induction; supportive evidence (S)	Pharmacological NRF2 activation reduced cytotoxicity and apoptosis	Reduced manganese-induced cell death	Tumour-derived cell line; tBHQ is not NRF2-specific and no knockdown or intact-animal confirmation was performed

Abbreviations: ARE, antioxidant-response element; CdCl_2_, cadmium chloride; GCLC, glutamate–cysteine ligase catalytic subunit; GPX3, glutathione peroxidase 3; GSH, reduced glutathione; GSSG, oxidised glutathione; HMOX1, haem oxygenase 1; MnCl_2_, manganese chloride; NQO1, NAD(P)H quinone dehydrogenase 1; NRF2, nuclear factor erythroid 2-related factor 2; PPARγ, peroxisome proliferator-activated receptor gamma; ROS, reactive oxygen species; SOD, superoxide dismutase; tBHQ, tert-butylhydroquinone.

*Strength of NRF2 evidence:* D, direct dependence demonstrated by NRF2 knockdown, deletion or selective inhibition that altered the toxic or protective response; S, supportive functional evidence based on nuclear translocation, ARE binding or target-gene induction, but without selective loss-of-function confirmation; I, involvement inferred from NRF2 abundance or immunoreactivity alone. Increased NRF2 expression without downstream functional evidence was not considered proof of effective antioxidant protection.

## 5 Convergent molecular mechanisms

Although cadmium, lead, mercury, arsenic and manganese differ in their chemical properties, toxicokinetics and primary molecular targets, their neurotoxic effects converge on several interconnected molecular pathways. Prominent among these are oxidative and electrophilic stress, glutathione depletion, mitochondrial dysfunction, disturbed calcium homeostasis and neuroinflammatory signalling. These processes can interact with impaired proteostasis, lipid peroxidation, altered synaptic and neurotrophic signalling and regulated cell-death pathways. NRF2 lies at an important interface within this network because it coordinates antioxidant, glutathione-dependent and mitochondrial defences while interacting with inflammatory and metabolic signalling. Thus, rather than operating as isolated mechanisms, redox imbalance, mitochondrial dysfunction and inflammation form a closely interconnected axis through which distinct environmental metals and metalloids can ultimately produce synaptic dysfunction, neuronal injury and functional neurological impairment. Reactive oxygen species may increase through mitochondrial electron leakage, inhibition of antioxidant enzymes, displacement of essential metals or mobilisation of redox-active iron and copper (Cheng et al., 2021). Some metals also stimulate nitric oxide production through inflammatory and calcium-dependent pathways. Then nitric oxide can react with superoxide to generate peroxynitrite, a potent oxidant that damages proteins, lipids and mitochondrial respiratory complexes (Halliwell, 2006). Thus, oxidative and nitrosative stress often reinforce each other rather than acting as separate mechanisms.

Glutathione is a major point of convergence between metal-induced oxidative injury and NRF2-dependent defence. Metals and their metabolites may bind directly to glutathione sulfhydryl groups, consume GSH during conjugation and detoxification, or interfere with enzymes involved in its synthesis and recycling (Tchounwou et al., 2012). NRF2 activation can counter these effects through induction of genes involved in glutathione synthesis and redox maintenance, including *GCLC* and *GCLM* (Hayes and Dinkova-Kostova, 2014). However, transcriptional induction does not necessarily translate into restoration of the reduced glutathione pool. During continuing metal exposure, direct thiol binding, peroxide detoxification and persistent mitochondrial oxidant production may consume GSH faster than it can be synthesised or regenerated. Oxidation of GSH generates GSSG, and failure to efficiently recycle GSSG back to GSH shifts the GSH/GSSG balance towards a more oxidising intracellular environment. Thus, NRF2 nuclear activation and target-gene induction can coexist with persistent redox failure when the magnitude of toxicant-driven glutathione consumption exceeds the capacity of the compensatory response. This pattern is illustrated by methylmercury-exposed microglia, in which NRF2 nuclear translocation and induction of *Hmox1*, *Nqo1* and *Slc7a11* occurred alongside increased oxidant production and a reduced GSH/GSSG ratio (Ni et al., 2010).

This distinction is particularly important in the nervous system because neurons possess comparatively limited intrinsic antioxidant capacity and depend partly on astrocytic provision of glutathione precursors (Shih et al., 2003). Persistent GSH depletion limits glutathione-peroxidase-dependent peroxide removal, promotes oxidation of protein thiols and membrane lipids, and increases mitochondrial vulnerability. Mitochondrial injury can in turn generate additional reactive oxygen species, establishing a feed-forward cycle of glutathione consumption, oxidative damage and bioenergetic failure. Accordingly, coordinated NRF2 target-gene induction without recovery of the GSH/GSSG balance should be interpreted as pathway engagement with incomplete functional redox recovery rather than as definitive evidence of effective neuroprotection. Restoration of GSH/GSSG together with improved mitochondrial and neurological outcomes provides stronger evidence of biologically effective NRF2-mediated defence. Nevertheless, GSH/GSSG should not be considered the sole criterion for neuroprotection because NRF2 also supports thioredoxin-dependent defence, detoxification, mitochondrial metabolism and inflammatory regulation.

Loss of glutathione defence encourages lipid peroxidation. This is particularly important in the brain because neuronal membranes contain large amounts of polyunsaturated fatty acids (Cobley et al., 2018). Once initiated, lipid oxidation can spread through membranes and generate reactive aldehydes that modify proteins involved in ion transport, synaptic signalling and mitochondrial function (Halliwell, 2006). F_2_-isoprostanes provide relatively stable markers of lipid peroxidation but are most informative when interpreted alongside glutathione and mitochondrial outcomes (Montuschi et al., 2004).

Mitochondria are both targets and amplifiers of metal toxicity. Metals can inhibit respiratory-chain complexes, disturb substrate oxidation and reduce electron-transfer-system capacity. This leads to higher reactive oxygen species from the electron leak and reduced ATP production because of defective oxidative phosphorylation (Cheng et al., 2021). ATP depletion is especially harmful to neurons because action potentials, neurotransmitter recycling, axonal transport and ion gradients all require a continuous energy supply (Cobley et al., 2018). Damage to the mitochondrial membrane may cause loss of membrane potential, swelling and opening of the mitochondrial permeability transition pore. Cytochrome c can subsequently enter the cytoplasm and initiate apoptosis (Qu et al., 2020).

Calcium dysregulation can amplify mitochondrial injury. Methylmercury-induced mitochondrial dysfunction in rat striatal synaptosomes was calcium-dependent (Dreiem and Seegal, 2007), while cadmium altered mitochondrial dynamics through calcium-dependent recruitment of Drp1 in neuronal models (Xu et al., 2016). These findings link disrupted calcium handling to metal-induced bioenergetic failure.

Mitochondrial injury is also connected to neuroinflammation. Manganese-associated disruption of astrocytic mitochondrial dynamics was accompanied by inflammatory signalling (Sarkar et al., 2018). In cadmium models, NRF2 deficiency increased microglial activation and synaptic engulfment (Li et al., 2025). NRF2 can restrain brain inflammation (Innamorato et al., 2008), and its reciprocal interaction with NF-κB depends on cellular context (Wardyn et al., 2015).

Mitochondrial permeabilisation can promote apoptosis (Qu et al., 2020), whereas ferroptosis should only be inferred when lipid peroxidation is accompanied by appropriate evidence of GPX4 impairment, iron dependence and pharmacological rescue (Song and Long, 2020; Sahoo and Sharma, 2023).

NRF2 also interacts with PGC-1α, a regulator of mitochondrial biogenesis and energy metabolism. Cooperation between these pathways can support antioxidant defence and the replacement of damaged mitochondria (Gureev et al., 2019). Mitochondrial quality control further depends on fission, fusion and mitophagy, including PINK1-Parkin-mediated recognition and removal of damaged organelles (Pickles et al., 2018). When these processes fail, dysfunctional mitochondria accumulate and continue producing oxidants. These interactions link NRF2 signalling to mitochondrial quality control.

These mechanisms indicate that metal neurotoxicity cannot be attributed to oxidative stress alone but reflects an interconnected failure of redox regulation, mitochondrial bioenergetics and inflammatory control. This is summarised below in Figure 2.

**FIGURE 2:**
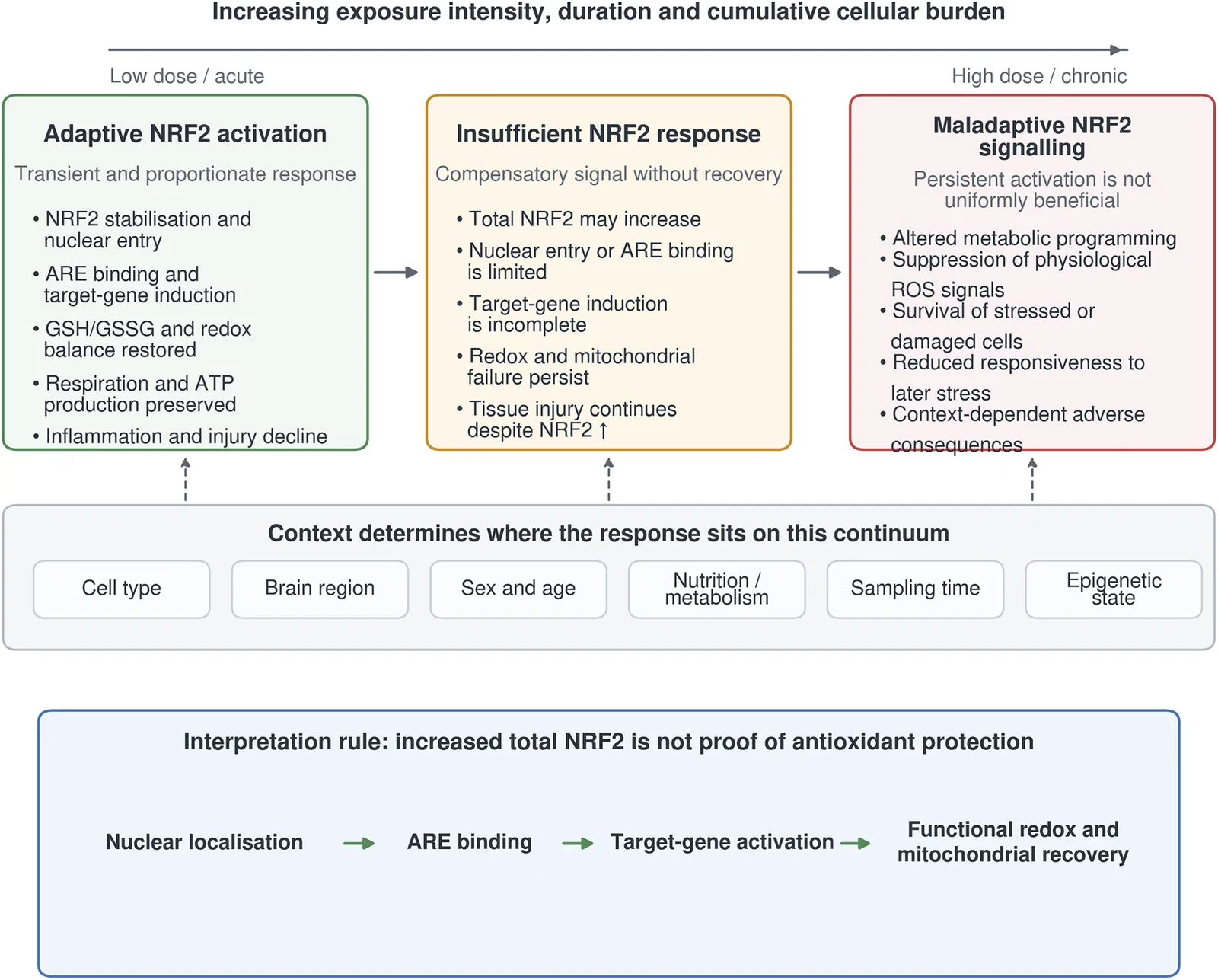
Convergent pathways linking environmental metal exposure to neuronal injury. Environmental metals promote reactive oxygen and nitrogen species, glutathione depletion, lipid peroxidation, calcium dysregulation and mitochondrial respiratory impairment. Reduced ATP production, mitochondrial membrane damage and defective mitophagy amplify oxidant formation and activate microglial and astrocytic inflammatory responses. NRF2 is shown as a conditional protective checkpoint interacting with NF-κB, PGC-1α and mitochondrial quality-control pathways. The figure distinguishes increased NRF2 expression from demonstrated functional activation, which requires nuclear translocation, antioxidant-response-element binding, target-gene induction and measurable functional recovery.

## 6 Context-dependent NRF2 responses

NRF2 responses are better viewed as a continuum than as a simple on-off event. After metal exposure, an increase in total NRF2 may represent an early attempt to restore cellular balance rather than evidence that protection has been achieved (Dodson et al., 2019). During brief, low-level exposure, NRF2 stabilisation may be followed by nuclear entry, antioxidant-response-element binding, target-gene induction and recovery of glutathione and mitochondrial function. As the dose or duration increases, however, oxidant production, thiol depletion and mitochondrial damage may exceed the capacity of this response. NRF2 may therefore increase while redox imbalance and tissue injury continue (Xiong et al., 2021). The timing and duration of activation are consequently as important as its magnitude (Dodson et al., 2019). For operational purposes, we propose that NRF2 responses be distinguished by combining evidence of pathway engagement with functional and, where possible, causal outcomes. Adaptive NRF2 activation should require evidence of pathway engagement, such as NRF2 nuclear accumulation and antioxidant-response-element binding, accompanied by coordinated induction of downstream targets and measurable improvement or preservation of redox balance, mitochondrial function or neurological outcome (Yamamoto et al., 2018; Dodson et al., 2019). Evidence that genetic or pharmacological NRF2 inhibition attenuates this protection further strengthens classification as adaptive, as demonstrated in experimental models in which NRF2 knockdown, deletion or inhibition modifies the protective response (Ni et al., 2010; Ye et al., 2016; Li et al., 2025).

A compensatory-but-ineffective NRF2 response occurs when NRF2 is demonstrably activated, including nuclear signalling and/or downstream target-gene induction, but oxidative stress, glutathione depletion, mitochondrial dysfunction, inflammation or neuronal injury remains unresolved. Experimental observations of persistent NRF2 activation despite continuing oxidative or neurological injury illustrate this distinction (Tang et al., 2019; Xiong et al., 2021). In this setting, NRF2 activation can be interpreted as an endogenous attempt to counter cellular stress that is quantitatively or temporally insufficient to restore homeostasis.

By contrast, maladaptive NRF2 signalling should be reserved for situations in which persistent or excessive NRF2 activity itself contributes causally to an adverse phenotype. Prolonged NRF2 activation can alter cellular metabolism, suppress physiological redox signalling or support survival of damaged cells, although evidence for these effects is considerably stronger in tumour biology than in chronically metal-exposed nervous tissue (Robertson et al., 2020). Accordingly, classification as maladaptive should require causal evidence, such as improvement of the adverse phenotype after NRF2 suppression or worsening following sustained or excessive NRF2 activation. Persistent NRF2 expression in the presence of injury, without such causal evidence, should therefore be classified as compensatory-but-ineffective rather than maladaptive. These categories should additionally be interpreted in relation to exposure dose and duration, sampling time, brain region and cell type (Dodson et al., 2019; Bell et al., 2015; Levings et al., 2023).

The cellular location of NRF2 is especially important. Measurement of whole-tissue or total-cell NRF2 cannot establish whether the protein reached the nucleus or initiated its transcriptional programme (Yamamoto et al., 2018). Cytoplasmic accumulation, altered degradation or a short-lived nuclear signal may produce an apparent increase without sustained DNA binding. Tissue-collection time can also influence interpretation: an early sample may capture adaptive signalling before recovery occurs, whereas a later sample may show persistent NRF2 expression alongside established injury (Dodson et al., 2019; Xiong et al., 2021). Studies should therefore combine total and nuclear NRF2 measurements with direct assessment of antioxidant-response-element occupancy, expression of relevant target genes and functional endpoints collected at biologically informative times.

The interpretation of HMOX1 requires particular caution. Although HMOX1 is frequently used as a downstream marker of NRF2 activity, its transcription is not exclusively determined by NRF2 and is strongly influenced by BACH1 and other stress-responsive regulatory mechanisms (Reichard et al., 2007; Medina et al., 2020). Consequently, increased HMOX1 expression alone should not be regarded as definitive evidence of functional NRF2 transcriptional activation. More robust assessment requires convergent evidence of NRF2 nuclear localisation and/or ARE-dependent transcription together with coordinated induction of multiple established NRF2-responsive genes, such as NQO1, GCLC, GCLM and TXNRD1. Even these molecular measures primarily establish pathway engagement; claims of functional NRF2-mediated protection should additionally be supported by restoration or preservation of relevant redox, mitochondrial or neurological outcomes and, where feasible, by genetic or pharmacological manipulation of NRF2.

The response also differs among brain cells. Mature neurons generally have lower NRF2 activity than astrocytes and other supporting cells, partly because neuronal *NFE2L2* expression is epigenetically restrained (Bell et al., 2015; Levings et al., 2023). An increase measured in whole-brain tissue may therefore arise mainly from astrocytes or activated microglia and may not indicate that vulnerable neurons are protected. Regional differences in metal accumulation, cellular composition, energy demand and inflammatory activity may also produce distinct NRF2 responses in the hippocampus, cortex, cerebellum and basal ganglia.

Age, sex, nutritional and metabolic status can alter basal NRF2 activity, the threshold for pathway activation and the ability to translate target-gene induction into functional protection (Schmidlin et al., 2019).

Epigenetic regulation can modify basal NFE2L2 expression and its inducibility through DNA methylation, histone modification and changes in chromatin accessibility (Bell et al., 2015; Levings et al., 2023). Prolonged NRF2 activity is not necessarily associated with progressively greater protection. Physiological reactive oxygen species contribute to redox-sensitive signalling, mitochondrial regulation and neuronal processes involved in synaptic plasticity and memory; consequently, sustained enhancement of antioxidant capacity could theoretically attenuate signalling that depends on tightly controlled oxidant production (Massaad and Klann, 2011; Heurtaux et al., 2022). Persistent NRF2 activity may also alter cellular metabolic programming by promoting antioxidant, detoxification and NADPH-generating pathways and may increase the capacity of stressed cells to survive despite persistent molecular or mitochondrial damage. Such effects are well recognised as potentially advantageous to damaged or transformed cells in other biological contexts (Robertson et al., 2020). During prolonged exposure to toxic metals and metalloids, continuous NRF2 activation has also been implicated in cellular adaptation and mechanisms associated with metal-induced carcinogenesis (Buha et al., 2021; Tripathi et al., 2024). However, direct evidence that persistent NRF2 activation itself causes neuronal injury during chronic metal exposure remains limited. In the CNS, possible consequences—including excessive suppression of physiological redox signalling, altered metabolic adaptation and persistence of dysfunctional cells—should therefore be considered mechanistically plausible rather than established outcomes. Accordingly, sustained NRF2 signalling should be classified as maladaptive only when persistent pathway activation can be linked to an adverse functional phenotype, preferably through causal experimental evidence.

These context-dependent transitions are summarised in Figure 3.

**FIGURE 3:**
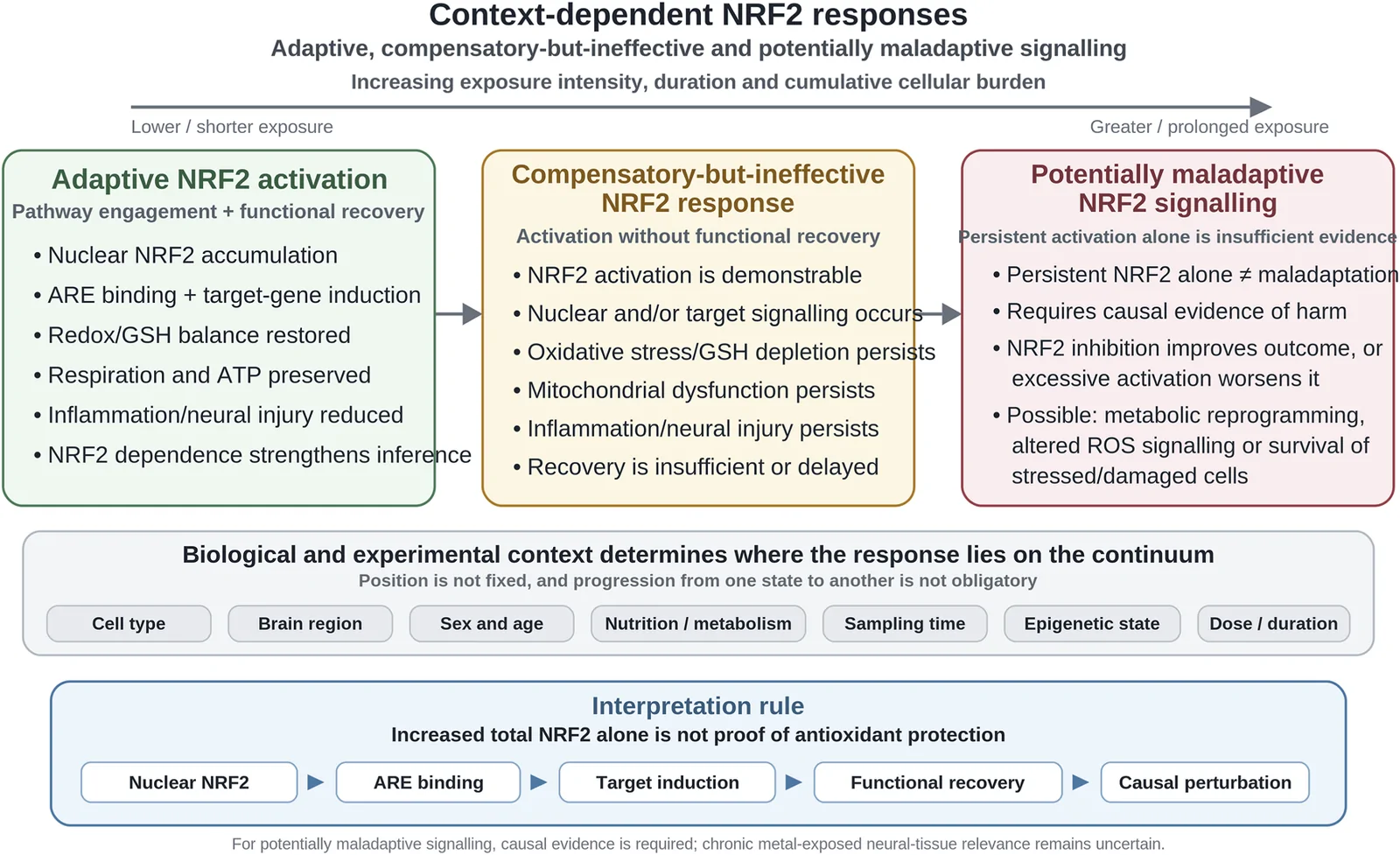
Context-dependent transition from adaptive NRF2 activation to compensatory-but-ineffective and potentially maladaptive NRF2 signalling. NRF2 responses to environmental metal and metalloid exposure occur along a continuum influenced by exposure intensity, duration and cumulative cellular burden. During lower-level or acute stress, adaptive NRF2 activation is characterised by NRF2 stabilisation and nuclear translocation, antioxidant-response-element (ARE) binding, coordinated induction of cytoprotective target genes, and measurable restoration or preservation of redox balance, mitochondrial function and cellular integrity. With increasing or prolonged exposure, NRF2 may remain elevated despite inadequate nuclear signalling, incomplete target-gene induction and persistent oxidative, mitochondrial, inflammatory or neuronal injury, representing a compensatory-but-ineffective response. Persistent or excessive NRF2 activity may become potentially maladaptive when it contributes to altered metabolic programming, suppression of physiological oxidant signalling or survival of damaged cells; however, such classification requires functional and preferably causal evidence rather than NRF2 abundance alone. Cell type, brain region, sex, age, nutritional and metabolic status, sampling time and epigenetic state can shift the position of the response along this continuum. Accordingly, increased total NRF2 should not be interpreted as evidence of neuroprotection without confirmation of nuclear localisation, ARE-dependent transcriptional activity and functional recovery.

## 7 Pharmacological modulation of NRF2

Pharmacological activation of NRF2 is attractive because it can strengthen several antioxidant and metabolic defences simultaneously. Nevertheless, an “NRF2 activator” is not necessarily an NRF2-selective drug. Most established activators are electrophiles that react with sensor cysteines on KEAP1, reduce NRF2 degradation and thereby activate the pathway indirectly (Yamamoto et al., 2018). Because these compounds may also modify cysteines in other proteins, their anti-inflammatory or mitochondrial effects cannot automatically be attributed to NRF2 (Schulze-Topphoff et al., 2016). Newer non-electrophilic compounds that interrupt the KEAP1-NRF2 protein interaction may provide greater selectivity, although their application in metal-induced neurotoxicity remains limited (Dodson et al., 2019).

Dimethyl fumarate has substantial clinical experience in multiple sclerosis (Gold et al., 2022). It is rapidly converted to monomethyl fumarate and promotes NRF2 stabilisation partly through modification of KEAP1 cysteines (Brennan et al., 2015). In cultured cells, dimethyl fumarate produced NRF2 nuclear translocation and target-gene induction but also caused an early, concentration-dependent decrease in glutathione before recovery occurred (Brennan et al., 2015). The biphasic response emphasizes the importance of dose and time of sampling. Dimethyl fumarate may promote mitochondrial biogenesis via an NRF2-dependent mechanism (Hayashi et al., 2017), yet some of its immunomodulatory effects remain intact in Nrf2-null animals (Schulze-Topphoff et al., 2016). These NRF2-independent effects require separate mechanistic evaluation (Schulze-Topphoff et al., 2016). Consequently, increased NRF2 expression or loss of protection following ML385 treatment provides useful evidence, but neither observation alone establishes that every effect of dimethyl fumarate is mediated by NRF2.

Sulforaphane is an isothiocyanate that activates the KEAP1–NRF2 pathway. In mice exposed to methylmercury, isothiocyanate treatment reduced methylmercury accumulation through an NRF2-dependent increase in cellular export mechanisms, demonstrating that NRF2 activation can sometimes influence toxicokinetics as well as tissue defence (Toyama et al., 2011). The effects observed with a defined experimental preparation should not be assumed to apply to unstandardised dietary intake.

Bardoxolone methyl potently activates NRF2 but has limited evidence in metal neurotoxicity and substantial off-target activity. The early termination of the BEACON trial because of excess cardiovascular events illustrates why strong NRF2 activation does not guarantee an acceptable therapeutic window (de Zeeuw et al., 2013).

Curcumin and related phytochemicals can increase NRF2 signalling and reduce metal-induced injury, but their multiple molecular targets, direct antioxidant effects and variable bioavailability make NRF2-specific attribution difficult (Yang et al., 2019).

NRF2 dependence can be tested pharmacologically using ML385. ML385 binds the Neh1 DNA-binding domain of NRF2 and interferes with binding of the NRF2-MAFG complex to antioxidant-response elements (Singh et al., 2016). If ML385 reverses an activator’s effects on NRF2 target genes and functional outcomes, NRF2 involvement becomes more plausible. However, ML385 was developed as an experimental probe rather than a clinically validated NRF2 antagonist (Singh et al., 2016). Appropriate vehicle and toxicity controls are essential, and pathway inhibition should be confirmed by measuring nuclear NRF2, DNA binding and target-gene expression.

Genetic deletion or knockdown provides complementary causal evidence. Whole-body deletion, however, may produce developmental compensation and systemic metabolic changes. Conditional deletion in neurons, astrocytes or microglia is therefore preferable when investigating cell-specific neuroprotection. Ideally, pharmacological inhibition, genetic manipulation and rescue experiments should produce consistent findings. Dose and timing should aim for proportionate pathway activation while comparing preventive with post-exposure schedules and monitoring toxicity.

Clinical evidence for dimethyl fumarate derives primarily from multiple sclerosis rather than metal-induced neurotoxicity. The ENDORSE study reported sustained effectiveness during long-term follow-up and characterised adverse events, including gastrointestinal effects and lymphopenia (Gold et al., 2022). In patients from the DEFINE and CONFIRM trials, dimethyl fumarate increased NQO1 expression, whereas HO1 induction did not reach statistical significance (Gopal et al., 2017). A separate study associated early NQO1 induction with subsequent disease-activity status (Hammer et al., 2018). Differences in immune-cell populations and transcriptional responses have also been associated with clinical response to dimethyl fumarate (Sánchez-Sanz et al., 2023). These observations support peripheral pathway engagement but do not establish that all clinical effects are NRF2-dependent.

Sulforaphane has been evaluated in a randomised trial in children with autism spectrum disorder, with results differing between clinician-rated and caregiver-rated outcomes (Ou et al., 2024). Such findings do not establish efficacy against metal-induced neurological injury. The excess cardiovascular events in the bardoxolone BEACON trial further show why antioxidant-pathway activation cannot substitute for clinical safety evaluation (de Zeeuw et al., 2013). These clinical studies do not test whether NRF2 activation prevents or reverses metal-induced neurotoxicity. Exposure-specific trials should therefore incorporate metal burden, pharmacodynamic markers, neurological outcomes and safety endpoints.

Finally, brain and blood metal concentrations should be measured alongside redox, mitochondrial and neurological outcomes. An intervention may reduce metal burden by altering absorption, binding or export (Toyama et al., 2011). Alternatively, it may leave tissue metal concentrations unchanged while increasing cellular tolerance. These are mechanistically different outcomes and should not be described interchangeably. The major interventions, evidence supporting NRF2 dependence and principal limitations are compared in Table 2.

**TABLE 2:** Pharmacological and experimental approaches used to investigate NRF2 signalling in metal-induced neurotoxicity.

Intervention/tool	Principal action	Metal-specific findings	Broader evidence/NRF2 dependence	Key limitations	Supporting references
Dimethyl fumarate (DMF)	Electrophilic KEAP1 modification; NRF2 activation	Metal-specific neuroprotection not established by the studies cited here	Non-metal models: NRF2 activation and mitochondrial biogenesis; immune effects can be NRF2-independent	GSH depletion; multiple targets; uncertain transfer to metal exposure	(Brennan et al., 2015; Hayashi et al., 2017; Schulze-Topphoff et al., 2016)
Sulforaphane	KEAP1 modification; NRF2 activation	MeHg: Reduced tissue Hg burden in mice; enhanced export in hepatocytes	Metal-specific: Nrf2-null comparisons support dependence of Hg-burden reduction	Clearance effects do not establish neuron-specific protection	(Toyama et al., 2011)
Bardoxolone methyl	Electrophilic NRF2 activator	No metal-exposure evidence in the cited clinical trial	Non-metal clinical evidence: Cardiovascular safety findings in chronic kidney disease	Cardiovascular toxicity; no proof of benefit in metal neurotoxicity	(de Zeeuw et al., 2013)
Curcumin	NRF2 activation; multiple molecular targets	MeHg-exposed rat astrocytes: Reduced oxidative injury and improved antioxidant defences	Metal-specific: NRF2 siRNA attenuated cytoprotection	*In vitro* evidence; limited bioavailability and target selectivity	(Yang et al., 2019)
Other electrophilic NRF2 activators (e.g., tBHQ)	Electrophilic stimulation of NRF2 signalling	tBHQ: Protection in lead-exposed neural models and manganese-exposed PC12 cells	Metal-specific: NRF2/HO-1 knockdown reduced tBHQ protection in lead-exposed SH-SY5Y cells	Preclinical; dependence varies by compound and exposure model	(Ye et al., 2016; Li et al., 2011)
ML385	Inhibits NRF2–MAFG binding to AREs	Metal-specific effects require validation in the exposure model	Non-metal evidence: NRF2 inhibition characterised in biochemical assays and lung-cancer models	Experimental probe; confirm target inhibition and off-target effects	(Singh et al., 2016)
NRF2 deletion/knockdown	Reduces or removes NRF2 expression	Tests NRF2 involvement in metal-induced injury and protection in experimental models	Metal-specific loss-of-function evidence; causal strength depends on the model and controls	Compensation; cell-type and systemic effects; limited human translation	(Ni et al., 2010; Ye et al., 2016; Li et al., 2025)

Authors’ synthesis of the cited studies. Metal-specific findings derive from metal-exposure models; broader evidence derives from other experimental or clinical settings and does not establish efficacy in metal-induced neurotoxicity. NRF2 dependence is reported only to the extent tested in the cited models. ARE, antioxidant response element; GSH, glutathione; MeHg, methylmercury; tBHQ, tert-butylhydroquinone.

## 8 Limitations and unresolved questions

The principal limitation is that evidence linking human metal exposure, NRF2 signalling and neurological outcomes remains incomplete. The human studies discussed above associate exposure biomarkers with cognitive or structural outcomes, but do not establish neural NRF2 dependence (Li et al., 2018; Reuben et al., 2017; Takeuchi et al., 2022). Peripheral molecular studies provide complementary but limited evidence. In a pilot study of 24 participants, associations between blood cadmium, mercury or lead and stress- or inflammation-related gene expression did not remain significant after correction for multiple testing (Monastero et al., 2018). Such measurements therefore do not demonstrate effective NRF2-mediated protection in the brain. Experimental studies also need to distinguish changes in total NRF2 or isolated target proteins from demonstrated DNA binding and causal NRF2 dependence.

Differences in exposure dose, duration, mixtures and host characteristics complicate translation from experimental models to human populations. Experimental findings obtained using high-dose tissue exposures are not directly equivalent to peripheral measurements during lower-level human exposure (Monastero et al., 2018). Co-exposure can also influence neurological outcomes, as illustrated by experimental metal-mixture studies (Rai et al., 2010) and human manganese–lead co-exposure research (Claus Henn et al., 2012). Inconsistent sampling times further complicate the distinction between early NRF2 adaptation and later pathway failure (Dodson et al., 2019).

Inadequate randomisation can bias animal experiments (Hirst et al., 2014), and transparent reporting of randomisation and blinding is a core ARRIVE 2.0 recommendation (Percie du Sert et al., 2020). Underrepresentation of female animals limits assessment of sex-dependent effects (Beery and Zucker, 2011). Whole-brain measurements also require caution because NRF2 expression differs markedly among neural cell types (Bell et al., 2015; Levings et al., 2023).

Publication bias may overestimate treatment efficacy (Sena et al., 2010). The principal limitations and corresponding research priorities are summarised in Table 3.

**TABLE 3:** Evidence gaps, interpretive consequences and recommended research methods.

Evidence gap	Interpretive consequence	Recommended study design or measurement
Limited human mechanistic evidence; reliance on total NRF2	Uncertain human relevance; abundance ≠ activation	Longitudinal human biomonitoring; validated peripheral markers; nuclear NRF2 in experimental models
No DNA-binding assessment	Transcriptional activity unconfirmed	ARE occupancy by ChIP-qPCR or equivalent assay
Incomplete target-gene panel	Isolated changes may be nonspecific	Coordinated NQO1, GCLC, GCLM and TXNRD1 assessment
No causal confirmation	NRF2 association only	NRF2 inhibition or knockdown; conditional deletion; rescue
Excessive metal doses	Limited environmental relevance	Biomonitoring-informed doses; chronic low-level exposure
Inconsistent exposure and treatment timing	Adaptation versus failure unresolved	Defined exposure durations; serial sampling; preventive and post-exposure schedules
Poor randomisation and blinding reporting	Selection and measurement bias	ARRIVE 2.0 reporting; preregistered allocation, exclusions and outcomes
Few female or aged animals	Sex and age effects uncertain	Both sexes; varied life stages; powered interaction analyses
Limited regional and cell-specific analysis	Opposing responses may be masked	Separate brain regions; cell-specific or spatial assays
Few metal-mixture studies	Limited relevance to co-exposure	Environmentally relevant mixtures and co-exposures
Potential publication bias	Efficacy may be overstated	Study registration; neutral findings; open protocols and data

## 9 Future research directions

Future studies should move from NRF2 association to causal dependence by combining nuclear localisation and ARE occupancy with coordinated target-gene induction, functional redox and mitochondrial outcomes, and genetic or pharmacological loss-of-function approaches. Brain metal burden should be measured concurrently to distinguish reduced exposure from increased tissue tolerance.

Experimental designs should better reflect human exposure through chronic low-dose and mixture models, inclusion of both sexes and different life stages, and regional or cell-specific analysis. Translation will require prospective human studies integrating metal biomonitoring, NRF2-related markers, neurological assessment and neuroimaging. Candidate peripheral measures, including NFE2L2 and downstream target-gene expression, glutathione status and lipid-oxidation products, should be evaluated as investigational biomarkers rather than assumed indicators of neural NRF2 activity. Their usefulness for risk assessment or treatment monitoring should be tested against longitudinal neurological outcomes, with attention to blood-cell composition and relevant exposure and lifestyle covariates. These are research priorities, not validated clinical applications.

## 10 Conclusion

Environmental metals and metalloids converge on glutathione disruption, mitochondrial dysfunction, oxidative injury and neuroinflammation despite differences in toxicokinetics and tissue distribution. NRF2 provides an important adaptive defence, but increased NRF2 abundance alone cannot establish successful neuroprotection. Functional activation requires evidence of nuclear signalling, coordinated target-gene induction and recovery of relevant redox, mitochondrial or neurological outcomes.

Current evidence for causal NRF2 dependence remains uneven and human mechanistic data are limited. Future studies should combine environmentally relevant exposure models with cell- and region-specific NRF2 assessment, functional endpoints and genetic or pharmacological loss-of-function approaches. Such evidence is necessary to distinguish effective NRF2-mediated protection from an insufficient response to continuing cellular injury.
