# Supplementary material for: NRF2 signalling in environmental metal and metalloid neurotoxicity: distinguishing stress responses from functional neuroprotection
Source: Front Toxicol. 2026 Oct 26;8:1988769. doi: 10.3389/ftox.2026.1988769 (PMC13744800; doi:10.3389/ftox.2026.1988769)
Supplement: Supplementary file 1 [file Table1.docx]

**NRF2 signalling in environmental metal and metalloid neurotoxicity: distinguishing stress responses from functional neuroprotection**

**Mesole Samuel Bolaji^1^, Yusuf Uthman Ademola^3^, Okpanachi Alfred Omachonu^2^**

1. **Human Anatomy Unit, School of Medicine and Health Sciences Eden University Lusaka**
2. **School of Medicine and Health sciences, Eden University Lusaka Zambia.**
3. **Department of Human Anatomy, School of Medicine and Health Science, Mulungushi University, Livingstone campus, Zambia**

**Corresponding Author: - Mesole Samuel Bolaji**

**Email: - ms361450@gmail.com**

**Table S1. Principal components and functional outcomes of NRF2 signalling in the nervous system**

| **Component or process** | **NRF2-related mechanism** | **Importance in the nervous system** | **Evidence supporting functional activation** |
| --- | --- | --- | --- |
| KEAP1-cullin-3 regulation | KEAP1 recruits NRF2 to the cullin-3 ubiquitin-ligase complex, leading to proteasomal degradation under resting conditions | Maintains low basal NRF2 activity while allowing a rapid response to oxidative or electrophilic stress | Reduced NRF2 ubiquitination, increased NRF2 stability or disruption of the KEAP1-NRF2 interaction |
| NRF2 stabilisation and nuclear translocation | Modification of reactive KEAP1 cysteine residues reduces NRF2 degradation, allowing NRF2 to accumulate and enter the nucleus | Initiates the cellular response to disturbances in redox balance | Increased nuclear NRF2 and an increased nuclear-to-cytoplasmic NRF2 ratio |
| Antioxidant-response-element binding | Nuclear NRF2 forms a complex with small MAF proteins and binds antioxidant-response elements in target-gene regulatory regions | Converts NRF2 accumulation into a transcriptionally active response | Chromatin immunoprecipitation, DNA-binding assays or antioxidant-response-element reporter activity |
| NQO1 | Catalyses the two-electron reduction of quinones, thereby limiting redox cycling and reactive-species formation | Protects neural cells from quinone-related oxidative injury | Increased NQO1 transcription, protein abundance and enzyme activity |
| GCLC and GCLM | Form the catalytic and modifier subunits of glutamate–cysteine ligase, which controls the rate-limiting step in glutathione synthesis | Supports glutathione production in neurons and glial cells | Increased GCLC and GCLM expression accompanied by increased glutathione or restoration of the GSH/GSSG ratio |
| HMOX1/HO-1 | Degrades pro-oxidant haem to biliverdin, carbon monoxide and iron | Can limit haem-related injury, although excessive activity may increase available iron | Increased HMOX1 transcription and HO-1 protein or activity; HO-1 alone should not be treated as complete evidence of NRF2 activation |
| TXNRD1 | Maintains thioredoxin in its reduced and active form | Supports protein-thiol repair, peroxide control and cellular redox balance | Increased TXNRD1 expression accompanied by improved thioredoxin-system activity |
| Glutathione synthesis and recycling | NRF2 promotes cystine uptake, glutathione synthesis, glutathione reductase activity and the production of NADPH required for glutathione recycling | Maintains the reduced glutathione pool and protects neural cells from oxidative injury | Increased glutathione, decreased oxidised glutathione and restoration of the GSH/GSSG ratio |
| Mitochondrial homeostasis | NRF2 supports respiratory-substrate availability, mitochondrial membrane potential, oxidative phosphorylation and ATP production | Maintains the high energy requirements of neurons and limits mitochondrial oxidant production | Improved mitochondrial respiration, membrane potential, respiratory coupling and ATP content |
| PGC-1α interaction | NRF2 and PGC-1α jointly support mitochondrial biogenesis, antioxidant defence and metabolic adaptation | Links redox control with mitochondrial number, function and replacement | Increased mitochondrial biogenesis markers, mitochondrial DNA content or respiratory capacity |
| Astrocytes | Astrocytes generally mount a strong NRF2 response and increase glutathione synthesis and release | Astrocyte-derived glutathione and its precursors provide indirect antioxidant support to neighbouring neurons | Increased astrocytic NRF2 activity, glutathione release and demonstrable protection of cocultured neurons |
| Neurons | Mature neurons generally have weaker intrinsic NRF2 activity, partly because NRF2 transcription is developmentally repressed | Makes neurons dependent on both their limited intrinsic defence and metabolic support from astrocytes | Demonstration of neuronal nuclear NRF2, target-gene induction and protection lost after NRF2 knockdown |
| Microglia and NF-κB | NRF2 limits oxidant production and can oppose NF-κB-dependent inflammatory signalling and cytokine transcription | Restrains excessive microglial activation and reduces inflammatory injury to neighbouring neurons | Reduced inflammatory mediators accompanied by NRF2 activation and reversal after NRF2 deletion or knockdown |
| p62, autophagy and GSK-3 | p62 can sequester KEAP1 and activate NRF2 during altered autophagy, whereas GSK-3 promotes KEAP1-independent NRF2 degradation | Connects NRF2 signalling with protein clearance, mitophagy and cellular energy status | Changes in autophagic flux or GSK-3 activity accompanied by altered NRF2 nuclear activity and target-gene expression |
| Overall functional confirmation | Functional NRF2 activity requires more than an increase in total NRF2 protein | Prevents compensatory or ineffective NRF2 accumulation from being misinterpreted as protection | Nuclear translocation, antioxidant-response-element occupancy, coordinated target induction, functional recovery and loss of protection after NRF2 inhibition or deletion |

**Abbreviations:** ARE, antioxidant-response element; ATP, adenosine triphosphate; GCLC, glutamate–cysteine ligase catalytic subunit; GCLM, glutamate–cysteine ligase modifier subunit; GSH, reduced glutathione; GSSG, oxidised glutathione; GSK-3, glycogen synthase kinase-3; HMOX1, haem oxygenase 1 gene; HO-1, haem oxygenase 1 protein; KEAP1, Kelch-like ECH-associated protein 1; NF-κB, nuclear factor kappa B; NQO1, NAD(P)H:quinone oxidoreductase 1; NRF2, nuclear factor erythroid 2-related factor 2; PGC-1α, peroxisome proliferator-activated receptor-γ coactivator 1α; TXNRD1, thioredoxin reductase 1.
